# Machine learning method based on radiomics help differentiate posterior pituitary tumors from pituitary neuroendocrine tumors and craniopharyngioma

**DOI:** 10.1038/s41598-025-05143-5

**Published:** 2025-06-06

**Authors:** Yukun Liu, Yanpeng Zhou, Chunyao Zhou, Zhenmin Wang, Ziwen Fan, Kai Tang, Siyuan Chen

**Affiliations:** 1 Department of Neurosurgery, Beijing Tiantan Hospital, Capital Medical University, Beijing, 100071 China; 2 Department of Neurosurgery, Air Force Medical Center, PLA, Beijing, 100142 China; 3 Department of Neurosurgery, Xiangya Hospital, Central South University, Changsha, 410008 Hunan China

**Keywords:** Machine learning, Radiomics, Posterior pituitary tumors, Predictive model, Diseases of the nervous system, Magnetic resonance imaging

## Abstract

Posterior pituitary tumors (PPTs) are rare neoplasms, but easily misdiagnosed as pituitary neuroendocrine tumor (PitNET) and craniopharyngioma. This study aimed to differentiate PPTs from PitNET and craniopharyngioma using a machine learning method based on radiomics. The cohort used for training and testing contained 33 PPTs and 99 non-posterior pituitary tumors (NPPTs). The validation cohort consisted of prospectively included patients (9 PPTs and 33 NPPTs). Radiomics features based on T1-weighted images and contrast-enhanced (CE) T1-weighted images were extracted, or both. Data of training and testing cohort were input to a nested 10-fold to build models, which were independently validated in the validation cohort. A least absolute shrinkage and selection operator (LASSO) was used for dimensionality reduction and random forest was used as classifier. Predictive models were successfully established, and models based on CE features had the best performance with an accuracy of 0.786, precision of 0.929, specificity of 0.778, sensitivity of 0.788, and area under the curve of 0.818 in validation. Nine features selected by more than 75% of the models based on CE features were identified as the most predictive features. We established a group of machine learning models to noninvasively differentiate PPTs from NPPTs before surgery, which may improve the surgical plan of PPTs to better complete resection of the tumors and protection of important structures around the tumors.

**Supplementary Information:**

The online version contains supplementary material available at 10.1038/s41598-025-05143-5.

## Introduction

Posterior pituitary tumor (PPT) is an entity defined by WHO classification of pituitary tumors in 2017, with diffuse nuclear expression of the homeobox transcription factor thyroid transcription factor-1 (TTF-1) as their immunohistochemical characteristic, including pituicytoma (PC), granular cell tumors of the neurohypophysis (GCT), spindle cell oncocytomas (SCO), and sellar ependymomas^1,2^. PPT is a rare disease, and most of the previous reports are case reports, without reliable population incidence statistics. Besides the rare incidence, it is extremely difficult to make a diagnosis preoperatively. In addition, the symptoms of PPT are similar to those of other common sellar tumors. PPT also lacks specific imaging features, thus it is easily misdiagnosed as PitNET and craniopharyngioma^3^. At present, the diagnosis of this disease mainly depends on the postoperative histological and immunohistochemical characteristics^1^ of specimens, and even intraoperative cytological examination is difficult to give the correct diagnosis^4^. The surgical resection is the gold standard for the PPT treatment, and most patients will be cured after total resection. However, the hypervascularity of this disease results in a high incidence of surgery-related bleeding (30.7% in a meta-analysis), which often leads to surgical interruption or incomplete resection of the tumor, and a high incidence of residual tumor (44.9%)^5^. Moreover, PPTs and PitNETs differ significantly in anatomical origin - PPTs originate from the posterior pituitary, whereas PitNETs arise from the anterior pituitary. This distinct origin results in critical differences in the spatial relationship between tumors and vital structures such as the pituitary stalk, optic nerves and chiasm, and cavernous sinus during surgery. Preoperative misidentification may lead to inadequate exposure planning, increasing risks of intraoperative injury to the visual pathways, neurovascular structures and postoperative endocrine dysfunction. Therefore, it is of great significance to distinguish it from other common tumors in sellar region before operation.

Previous studies have summarized the clinical and imaging features of these diseases. The locations of PPTs can be classified as intrasellar only, suprasellar only, and mixed intrasellar/suprasellar^6^. PCs and SCOs mainly occurred in the sellar/suprasellar region, while sellar GCTs are rare. SCOs often grow invasively, and 3% of SCOs have been reported to invade the cavernous sinus^7^. The signals of PCs are variable on MRI^8,9^, but most of them are reported to be isointensity on T1-weighted image, isointensity or hyperintensity on T2-weighted image, and homogeneous enhancement could be observed in most of the contrast-enhanced T1-weighted images. Most of them are solid with a sharp edge, and there are few lesions with cystic components and almost no calcification in it^6,10,11^. GCTs were reported isointensity on T1-weighted image, and isointensity or hypointensity on T2 -weighted image^12^. A ‘star-like crack’ feature had been found in some cases^13^. SCOs were reported isointensity on T1-weighted image, and isointensity or hyperintensity on T2 -weighted image, in addition to having hypointense millimetric foci and linear signal void areas on T1 and T2-weighted image^14^. For a systematic comparison of MRI characteristics across these tumor types, see Supplementary Table 1. Regarding the clinical manifestations of PPTs, 57.9% patients suffered from visual deficit, of which 45.9% presented with hypopituitarism. In addition, the proportion of patients with diabetes insipidus before surgery was very low^5^. Although PPTs have some clinical characteristics, it is still difficult to diagnose them correctly before surgery.

The artificial intelligence technology based on radiomics information has been widely used in the diagnosis of central nervous system tumors^15^. It is also applied in the differentiation diagnosis of sellar lesions such as PitNET and craniopharyngioma, to identify tumor types^16,17^ and subtypes^18–23^, predict molecular expression^24,25^, predict treatment response^26,27^, and predict tumor recurrence^28^. However, due to the large amount of data required, this method has not been applied to rare diseases.

The aim of this study is to utilize machine learning based on radiomics to construct a model to distinguish PPTs from PitNET and craniopharyngioma, which are difficult to differentiate from PPTs. This new approach will help assign surgical plans, complete tumor resection, and protect important structure of saddle region.

## Materials and methods

### Patient inclusion and criteria

The cohort used for training and testing contained 33 patients with PPTs who received primary surgical treatment between January 2008 and May 2021 at institutional database, 51 patients with PitNET and 48 patients with craniopharyngioma who received treatment between 2020 and 2021 at the same hospital. The validation cohort consisted of prospectively included 9 patients with PPTs, 17 patients with PitNET, and 16 patients with craniopharyngioma who received treatment from June 2021 to June 2022 at institutional database.

The diagnosis was confirmed by histopathological analysis of the surgically resected specimens. To be included in the study, patients had to have accessible preoperative sagittal T1-weighted and contrast-enhanced T1-weighted MRI data. Patients with concomitant neurosurgical space-occupying lesions (e.g., meningiomas, arachnoid cysts) or a history of prior sellar/parasellar interventions (including transsphenoidal surgery or craniotomy) were excluded to eliminate confounding from preexisting surgical alterations or coexisting pathologies. In each cohort, patients were divided into two subgroups based on histological diagnosis: posterior pituitary tumors (PPTs) and non-posterior pituitary tumors (NPPTs).

This study was approved by the Ethics Committee of Beijing Tiantan Hospital. Informed consent was obtained from all prospectively enrolled patients, and the need for informed consent of patients were included retrospectively was waived by the ethics committee. This study was conducted in accordance with the Declaration of Helsinki. The chi-squared test was used to assess the significance of performance differences based on certain patient characteristics. Student’s *t* test was used to assess differences in continuous variables extracted by different methods or from different types of images. The chi-squared test was used to compare the differences of sex and type of tumor between the different groups, while the Student’s t-test was used to compare the differences in age.

### Imaging data acquisition

MRI was scanned Magnetom Trio 3 T MR scanners (Siemens Healthcare, Erlangen, Germany). For T1-weighted images, the specifications were as follows: repetition time, 1200 ms; echo time, 11 ms; flip angle:120°; slice thickness, 5 mm; field of view, 220 mm × 220 mm; and matrix size, 256 × 256 pixels. For contrast-enhanced T1-weighted images, enhancement was achieved by injection of gadolinium-diethylenetriamine penta-acetic acid (Gd-DTPA; 0.1 mmol/kg, Beijing Beilu Pharmaceutical, Beijing, China) and the acquisition parameters were same as above.

T1-weighted images were registered to contrast enhanced images using FMRIB software library (FSL, https://fsl.fmrib.ox.ac.uk/fsl/fslwiki/)^29^. Lesions were manually segmented on sagittal contrast-enhanced T1-weighted images by two board-certified neurosurgeons using MRIcro (http://www.mccauslandcenter.sc.edu/mricro/*).* The segmentation was evaluated by a neuroradiologist with over 20 years of experience in brain tumor diagnosis. All three specialists were aware of the patients’ histopathology-based diagnosis.

### Extraction of radiomics information

An open-source Python package PyRadiomics (https://pyradiomics.readthedocs.io/) were used to extract radiomics data from T1-weighted and contrast-enhanced T1-weighted images. For each sequence of image, original radiomics features (including 14 shape features, 18 first-order features, 24 Gray level co-occurrence matrix (GLCM), 16 Gray level run length matrix (GLRLM) features, 16 Gray level size zone matrix (GLSZM) features and 14 Gray level dependence matrix (GLDM) features, and their derived features (including 264 log sigma features, 704 wavelet features, 88 square features, 88 square root features, 88 logarithm features, 88 exponential features, and 88 gradient features) were extracted from a total of 1510 features. The feature extraction used each sequence of image and was used to build models alone or in combination.

### Automated machine learning and statistical analysis

**Fig. 1:**
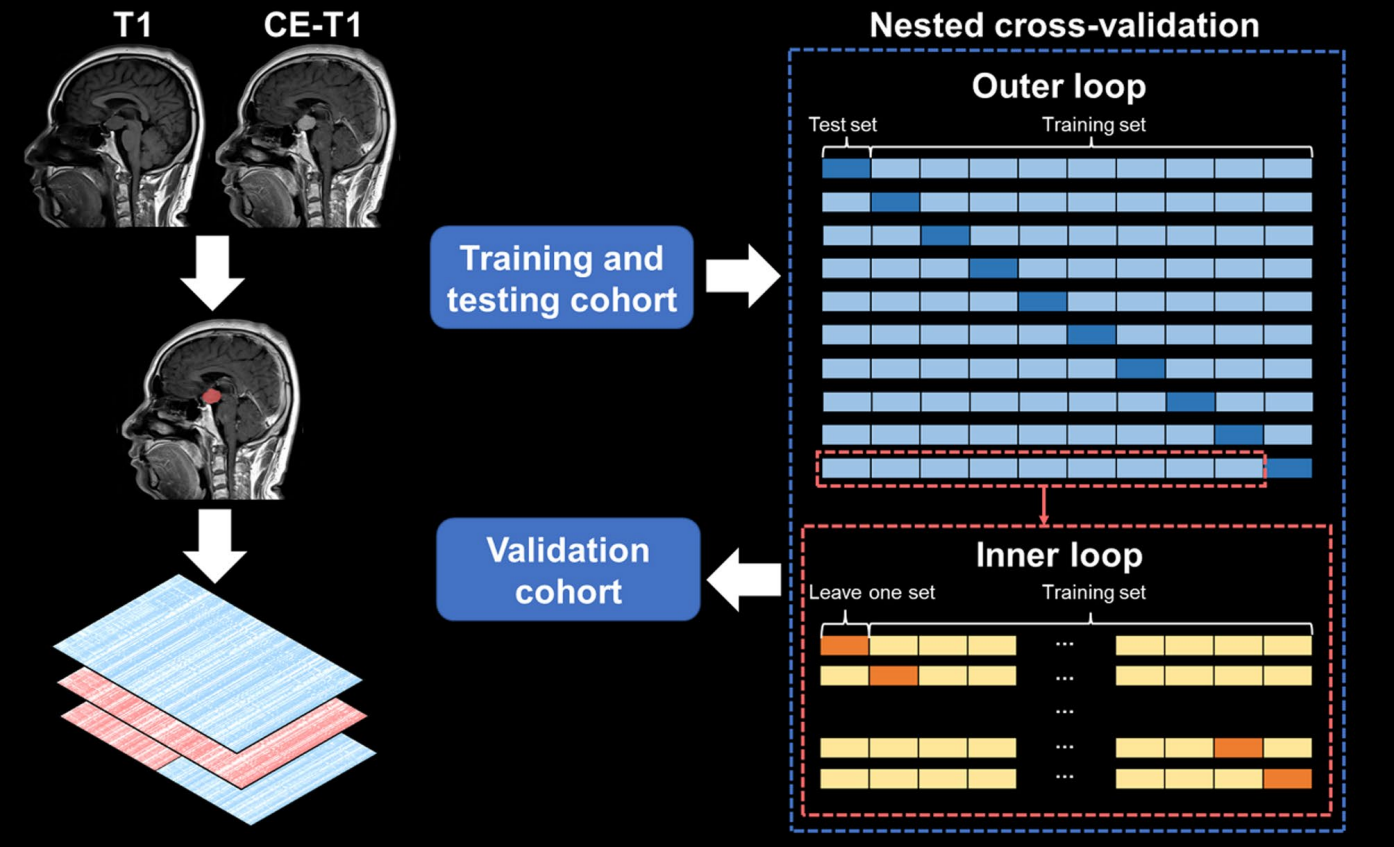
Workflow diagram. First, manual segmentation was performed on each patient’s contrast-enhanced (CE) T1-weighted images. Then, the radiomics features was extracted. Finally, the data of training and testing cohort were including in a nested cross-validation to build machine learning models which was used to validated in the validation cohort.

The analysis was performed using the Tree-based Pipeline Optimization Tool (TPOT), a Python library built upon scikit-learn. TPOT employs an iterative genetic algorithm to automatically optimize parameters. A nested cross-validation is used for model building, preliminary model prediction evaluation, and selection of the most predictive features. A 10-fold split were used to divide patients in training and testing cohort, and each fold is taken in turn as the testing set while the remaining as the training set. A least absolute shrinkage and selection operator (LASSO) was used to reduce dimension of features in each training set. Based on filtered features of training set, a random forest classifier was used to training predict models, and optimized the optimal configuration by a leave one out cross-validation (LOOCV) based on accuracy. Therefore, a model group of ten models will be generated for each data input. The prediction results of each trained optimal model in the corresponding testing set are accumulated, and finally used to evaluate the nested cross-validation prediction performance of the model groups. These groups of models were also used to predict the validation cohort, and the probability of prediction in each model was weighted to determine the final prediction. The input data of the best-performing model group in validation will be combined with clinical data, and the above process will be used to generate machine learning models and evaluate its predictive performance again. The features whose feature importance obtained by random forest algorithm was not zero and included by more than 75% models in the best predictive model group in validation are considered the most predictive features. The performance of model and group of models was assessed in terms of accuracy, recall, precision, sensitivity, specificity, and AUC. The workflow of this study was summarized in Fig. 1.

## Result

### Patient demographics

The cohort for training and testing consisted of 33 patients with PPT (15 men and 18 women, mean age of 48.0 ± 10.9 years) and 99 patients with NPPT (58 men and 41 women, mean age of 49.0 ± 10.5 years), while validation cohort consisted of 9 patients with PPT (4 men and 5 women, mean age of 44.0 ± 12.8 years) and 33 patients with NPPT (23 men and 10 women, mean age 43.0 ± 14.2 years). The clinical characteristics and distribution of the enrolled patients were summarized in Table 1. The two cohorts did not differ significantly in age and the ratio of the two sexes. There were no significant between-group differences in sex percentage and age between PPT and NPPT patients, but a significant difference was found in the incidence of abnormal pituitary hormone levels (*p* < 0.01). A quantitative comparison of eight pituitary hormones between the two groups is presented in Supplementary Table 2. Clinical information on histologic subgroups included in the PPT and NPPT groups was included in Supplementary Tables 3 and Supplementary Table 4.

**Table 1:** Distribution of clinical characteristics.

Clinical characteristics	Overall	PPTs (*n* = 42)	NPPTs (*n* = 132)	*p*	Training and testing (*n* = 132)	Validation (*n* = 42)	*p*
Age (year ± SD)	46.4 ± 11.5	46.4 ± 11.3	46.4 ± 11.5	0.98	47.1 ± 10.6	44.1 ± 13.8	0.14
Sex (n, ratio)				0.12			0.83
Male	97 (0.56)	19 (0.45)	78 (0.59)		73 (0.55)	26 (0.62)	
Female	77 (0.44)	23 (0.55)	54 (0.41)		59 (0.45)	16 (0.38)	
Pituitary hormone (n, ratio)				< 0.01*			0.86
Normal	60 (0.34)	22 (0.52)	38 (0.29)		46 (0.35)	14 (0.33)	
Abnormal	114 (0.66)	20 (0.48)	94 (0.71)		86 (0.65)	28 (0.67)	

PPTs, posterior pituitary tumors, NPPTs, non-posterior pituitary tumor.

### Optimal machine learning pipelines

**Fig. 2:**
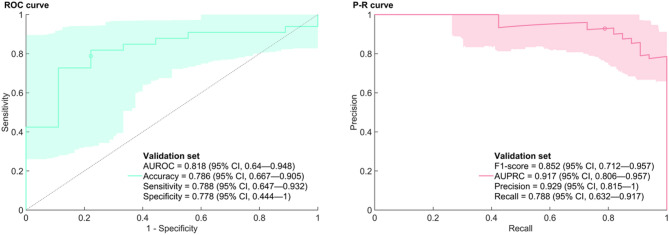
Receiver operating characteristic curve (ROC) and Precision-Recall (P-R) curve in the validation.

Ten models for each type of data input (T1-weighted, contrast-enhanced T1-weighted or both) were established to discriminate PPT and NPPT as a model group. The performance of these model group in validation and the nested cross-validation were summed in Table 2 and Supplementary Table 5, respectively. Among the PPTs in the validation cohort, 2 of 9 cases were misclassified: one PC (MR image provided in Supplementary Fig. 1) and one GCT. Model group based on contrast-enhanced T1-weighted features alone performed best in the validation cohort with accuracy of 0.786, precision of 0.929, specificity of 0.778, sensitivity of 0.788, and AUC of 0.818 (Fig. 2). The performance of model group based on T1-weighted features alone was poor in the nested cross-validation (AUC = 0.697) and even worse in validation cohort (AUC = 0.593). Performance of model group based on the combination of the two features described above (AUC = 0.803) was sightly inferior to that based on contrast-enhanced T1-weighted features alone in validation cohort. The clinical features were added to the best performing input data (contrasted-enhanced T1-weighted features alone) and the above modeling and validation were repeated. However, the clinical features were discarded in the dimension reduction process of each inner loop of nested cross-validation, so that the results were similar to those only used contrast-enhanced T1-weighted features as input and changed the random seed, so they would not be described in detail.

**Table 2:** The predicting performance of group of models in the validation cohort.

Features	Accuracy	Precision	Specificity	Sensitivity	AUC
T1-weighted	0.714	0.769	0.000	0.909	0.593
CE T1-weighted	0.786	0.929	0.778	0.788	0.818
T1-weighted & CE T1-weighted	0.750	0.929	0.778	0.743	0.803

CE, contrast-enhanced, AUC, area under curve.

### Key features selected in the best model group

**Fig. 3:**
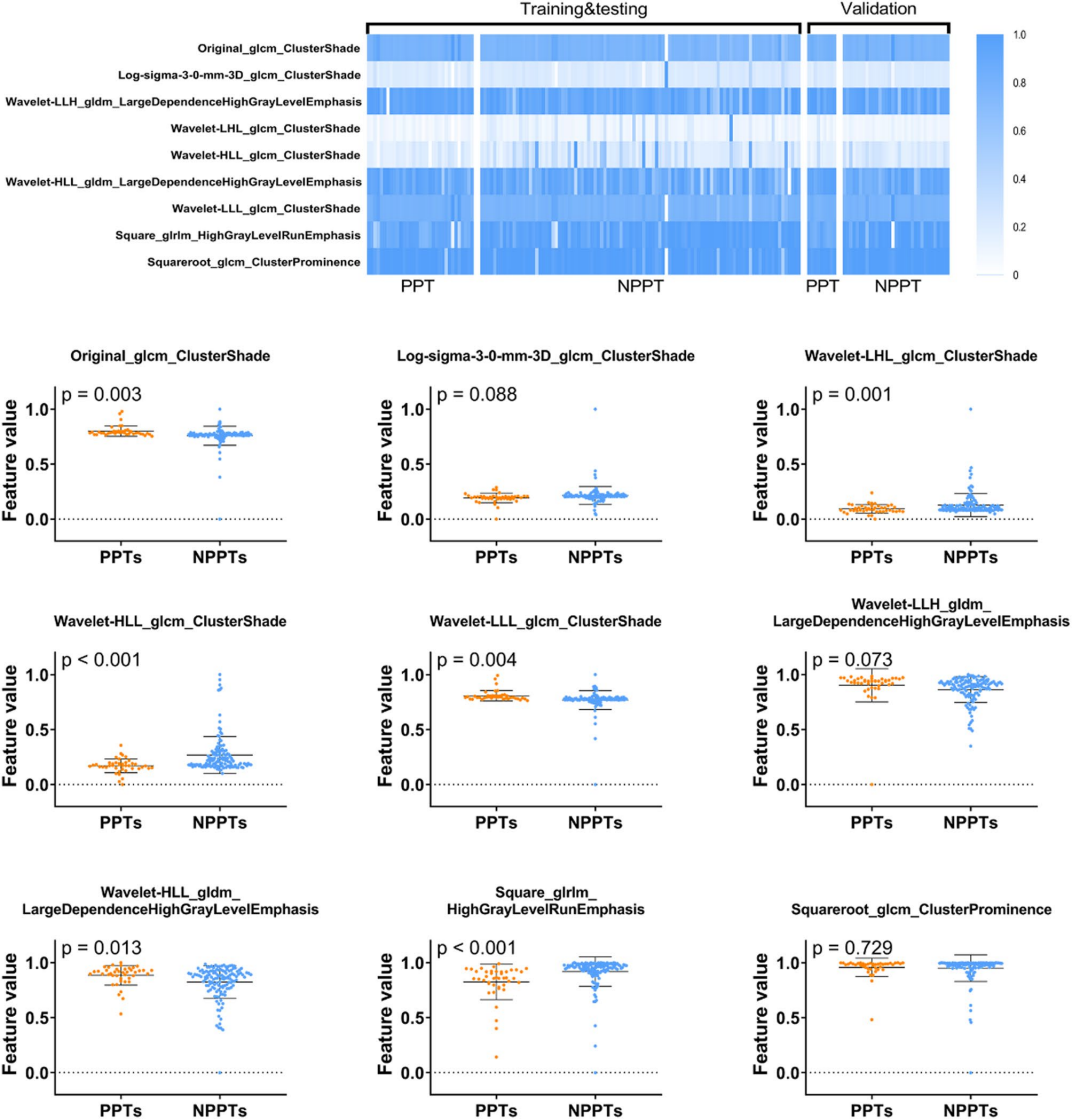
The most predictive features. The top half of the image is a heat map of the nine most predictive features. The values of the features were normalized using min–max normalization, and are represented by white to blue (0–1). In the bottom half of the image shows the difference between of these features between the posterior pituitary tumors (PPTs) and the non- posterior pituitary tumors (NPPTs).

There were nine features with non-zero importance of random forest classifier and were included in more than 75% of the 10 models based on contras-enhanced T1-weighted features: feature ClusterShade and four derived features of it, two derived features of LargeDependenceHighGrayLevelEmphasis, one derived feature of HighGrayLevelRunEmphasis, and one derived feature of ClusterProminence. These features were considered to be key features for distinguishing PPT and NPPT (Fig. 3).

## Discussion

In this study, radiomics features extracted from T1-weighted and contrast-enhanced T1-weighted image of PPTs, PitNETs, and craniopharyngiomas were used alone or in combination to build prediction models to discriminate these tumors. Models based on features extracted from contrast-enhanced T1-weighted images had the best predictive performance. Adding clinical information to this type of input did not improve the predictive performance of the model. Nine features were identified as the most important for the discrimination of PPTs and NPPTs in the nested cross-validation.

Although the imaging and clinical features of PPT, PitNET and craniopharyngioma had many similarities and were easy to be misdiagnosed, few studies explored to distinguish PPTs from NPPTs. Feng et al.^7^ suggested that most pituicytoma were located in the suprasellar midline, non-cystic and without calcification, and can be diagnosed in 90% of suitable cases based on imaging and clinical characteristics. However, this criterion was not specific enough and was not tested further in subsequent studies. Our radiomics based model achieved an accuracy of 0.811 in the nested cross-validation and 0.786 in the validation dataset. This suggested that our model was able to distinguish PPTs from NPPTs and exhibited a good robustness. Although clinical information did not significantly enhance the predictive performance of the models, PPTs and NPPTs were found to have differences in the incidence of abnormal pituitary hormone levels. These findings were consistent with the results reported by previous studies^5,30,31^. We believe that it is also an important distinguishing feature and has a certain suggestive role in clinical practice. More significant predictive value may be expected in future studies with larger sample sizes.

The models based on those features extracted from contrast-enhanced T1-weighted images had a better predictive performance than those models only based on T1-weighted features, suggesting that PPTs and NPPTs on contrast-enhanced images may have more significantly predictive values. Contrast-enhanced images of craniopharyngiomas have better discrimination, thus it generally manifests as a heterogenous mass with mixed cystic and solid multiloculated, and the contrast enhancement is present within solid components and as rim enhancement of the cystic areas^32^. PitNET presents a mild to moderate contrast enhancement, which is generally less than normal pituitary tissue. Most PPTs show homogeneous enhancement^5^ (especially in 80.5% of pituicytomas^3^) and are easily confused with PitNETs. This may explain the misclassification of one PC case in our cohort, which exhibited atypical imaging features: suprasellar extension was absent, and heterogeneous enhancement was observed. Radiomics features may provide discriminative power beyond that of human readers and most of the 9 key features were significantly different between the PPTs and NPPTs groups. For GLCM-based features, elevated Cluster Shade and its derived features suggest increased skewness in gray-level distribution, potentially linked to focal T1 hyperintensity and heterogeneous enhancement in PPTs. Elevated Cluster Prominence further reflected greater intensity variability in PPTs, possibly linked to histopathological heterogeneity. The Square feature derived from High Gray Level Run Emphasis was based on GLRLM, and indicated there were greater concentration of high gray-level values in the images of NPPTs. The two derived features of Large Dependence High Gray Level Emphasis were based on GLDM and indicated the joint distribution of large dependence with higher gray-level values was different between PPTs and NPPTs. Specifically, the elevated feature values in PPTs suggested clustered high-intensity voxels, potentially corresponding to cystic components or fluid accumulation observed on MRI. In contrast, NPPTs exhibited lower values due to more dispersed high-intensity regions. These subtle texture differences serve as the basis for discriminating PPTs and NPPTs.

Artificial intelligence based on radiomics has been successfully used in identifying confusing diseases in and around the saddle region. Zhao et al. used a logistics regression as a classifier to distinguish cystic-solid PitNET from craniopharyngioma, and could achieve an AUC of 0.88 in the test set^17^. Wang et al. used an artificial neural network to differentiate cystic PitNET from Rathke cleft cyst and with an AUC of 0.848, which was reported better than radiologist^16^. Serdar et al. used an support vector machines to discriminate hypophysitis and non-functioning PitNETs^23^. Zhang et al. used machine learning to discriminate 3 pairs of disease located in the anterior skull base with an AUC over 0.8 of each pair in test set, including PitNET/craniopharyngioma, meningioma/craniopharyngioma, and PitNET/Rathke cleft cyst^33^. These studies supported the feasibility of machine learning based on radiomics to identify diseases in the saddle region, and we are the first study to apply it to the diagnosis of posterior pituitary tumors.

There are still some limitations in this study. First, this study is a single-center study. As a rare disease, we hope that multi-center data can be included in the future to enhance the robustness of models. Second, the retrospective design inherently limited our MRI protocol selection. During the study period, our institutional standard for sellar tumor evaluation prioritized T1-weighted sequences with contrast enhancement for assessing tumor morphology and enhancement patterns. Sagittal T2-weighted images, which are critical for flow void detection in PPT diagnosis, were not systematically acquired in routine clinical protocols, which might have constrained our ability to fully exploit T2-derived diagnostic features. Third, we only discussed the differentiation between PPTs, PitNETs and craniopharyngiomas, while some other easily confused lesions, such as sellar meningiomas and pituitaritis, were not included in this study. We hope that more kinds of diseases can be included in the future to improve the usability of the models. Fourth, patients with more than one type of tumor were not included in this study. We excluded one pituicytoma patient complicated with PitNET. The co-occurrence of PitNETs and neuropituitary tumors is rare^34^, and these patients should be analyzed separately in the future. Finally, the tumor segmentation was performed by specialists with prior knowledge of the pathological diagnoses, and blinding was not implemented during this process, which may have introduced potential biases in imaging analysis.

We used machine learning method to bulid models to discriminate PPTs and NPPTs. Models based on CE features performed best with an accuracy of 0.786 and an AUC of 0.818 during validation. These models may be able to help the noninvasive diagnosis of PPTs before surgery, and to assist in the development of surgical plans to improve the probability of total resection and normal structural protection.

## Electronic supplementary material

Below is the link to the electronic supplementary material.

Supplementary Material 1

## Data Availability

The article’s data will be shared on reasonable request from the corresponding author.
