# Supplementary material for: Machine learning method based on radiomics help differentiate posterior pituitary tumors from pituitary neuroendocrine tumors and craniopharyngioma
Source: Sci Rep. 2025 Jun 6;15:19967. doi: 10.1038/s41598-025-05143-5 (PMC12144140; doi:10.1038/s41598-025-05143-5)
Supplement: Supplementary file 1 — Supplementary Material 1 [file 41598_2025_5143_MOESM1_ESM.docx]

**Supplementary Tables**

Supplementary Table 1. MRI characteristics of PPTs

| **Diseases** | **T1WI** | **T2WI** | **CE** | **Morphology** | **Other features** |
| --- | --- | --- | --- | --- | --- |
| **PCs** | Isointense | Isointense/Hyperintense | Homogeneous | Solid, sharp-edged | Rare cystic components; almost no calcification |
| **GCTs** | Isointense | Isointense/Hypointense |  |  | “Star-like crack” sign in some cases |
| **SCOs** | Isointense with hypointense foci | Isointense or hyperintense with linear signal voids |  |  | Hypointense millimetric foci and linear signal voids on both sequences |

Supplementary Table 2. Distribution of hormone level alterations in NPPT vs. PPT groups

|  | **NPPT** | | | **PPT** | | |
| --- | --- | --- | --- | --- | --- | --- |
|  | **Elevated** | **Decreased** | **Normal** | **Elevated** | **Decreased** | **Normal** |
| **PRL** | 56 | 3 | 71 | 10 | 0 | 32 |
| **GH** | 7 | 0 | 123 | 1 | 0 | 41 |
| **COR** | 0 | 0 | 129 | 0 | 0 | 42 |
| **LH** | 3 | 9 | 107 | 0 | 5 | 36 |
| **FSH** | 11 | 3 | 105 | 0 | 1 | 40 |
| **E2** | 6 | 0 | 113 | 0 | 0 | 41 |
| **P4** | 0 | 40 | 79 | 0 | 8 | 33 |
| **T** | 0 | 1 | 118 | 0 | 2 | 39 |

PRL, prolactin, GH, growth hormone, COR, cortisol, LH, luteinizing hormone, FSH, follicle stimulating hormone, E2, estradiol, P4, progesterone, T, testosterone.

In the NPPT group (n=132), one patient had no hormone results available, one patient presented hormone data exclusively for GH, and another patient lacked results for both GH and COR. Additionally, 11 patients showed hormone measurements limited to PRL, GH, and COR, with no other parameters recorded. Similarly, in the PPT group (n=42), a single patient exhibited hormone results restricted to PRL, GH, and COR, and no further measurements were available.

Supplementary Table 3. Clinical characteristics of posterior pituitary tumor, PitNET and Craniopharyngioma.

| Clinical characteristics | Posterior pituitary tumor  (n=42) | PitNET  (n=68) | Craniopharyngioma  (n=64) | p |
| --- | --- | --- | --- | --- |
| Age (year ± SD) | 46.43 ± 11.34 | 46.50 ± 11.05 | 48.50 ± 12.01 | 0.58 |
| Sex (n, ratio) |  |  |  |  |
| Male | 19 (0.45) | 39 (0.57) | 39 (0.61) | 0.27 |
| Female | 23 (0.55) | 29 (0.43) | 25 (0.39) |  |
| Pituitary hormone (n, ratio) |  |  |  | <0.01* |
| Normal | 22 (0.52) | 23 (0.34) | 15 (0.23) |  |
| Abnormal | 20 (0.48) | 45 (0.66) | 49 (0.77) |  |

Supplementary Table 4. Clinical characteristics of pituicytoma, granulosa cell tumor spindle cell oncocytoma.

| Clinical characteristics | Pituicytoma  (n=29) | | Granulosa cell tumor  (n=9) | | Spindle cell oncocytoma  (n=4) | |
| --- | --- | --- | --- | --- | --- | --- |
| Age (year ± SD) | 50.00 ± 10.27 | | 46.00 ± 11.15 | | 42.00 ± 19.40 | |
| Sex (n, ratio) |  | |  | |  | |
| Male | 13 (0.45) | | 4 (0.44) | | 2 (0.50) | |
| Female | 16 (0.55) | | 5 (0.56) | | 2 (0.50) | |
| Pituitary Hormone (n,ratio) | |  | |  | |  |
| Normal | 17 (0.59) | | 3 (0.33) | | 2 (0.50) | |
| Abnormal | 12 (0.41) | | 6 (0.67) | | 2 (0.50) | |

Supplementary Table 5. The predicting performance of group of models in the nested cross-validation.

| Features | Accuracy | Precision | Specificity | Sensitivity | AUC |
| --- | --- | --- | --- | --- | --- |
| T1-weighted | 0.750 | 0.800 | 0.333 | 0.889 | 0.697 |
| CE T1-weighted | 0.811 | 0.849 | 0.515 | 0.909 | 0.846 |
| T1-weighted & CE T1-weighted | 0.832 | 0.848 | 0.528 | 0.941 | 0.843 |

CE, contrast-enhanced, AUC, area under curve.


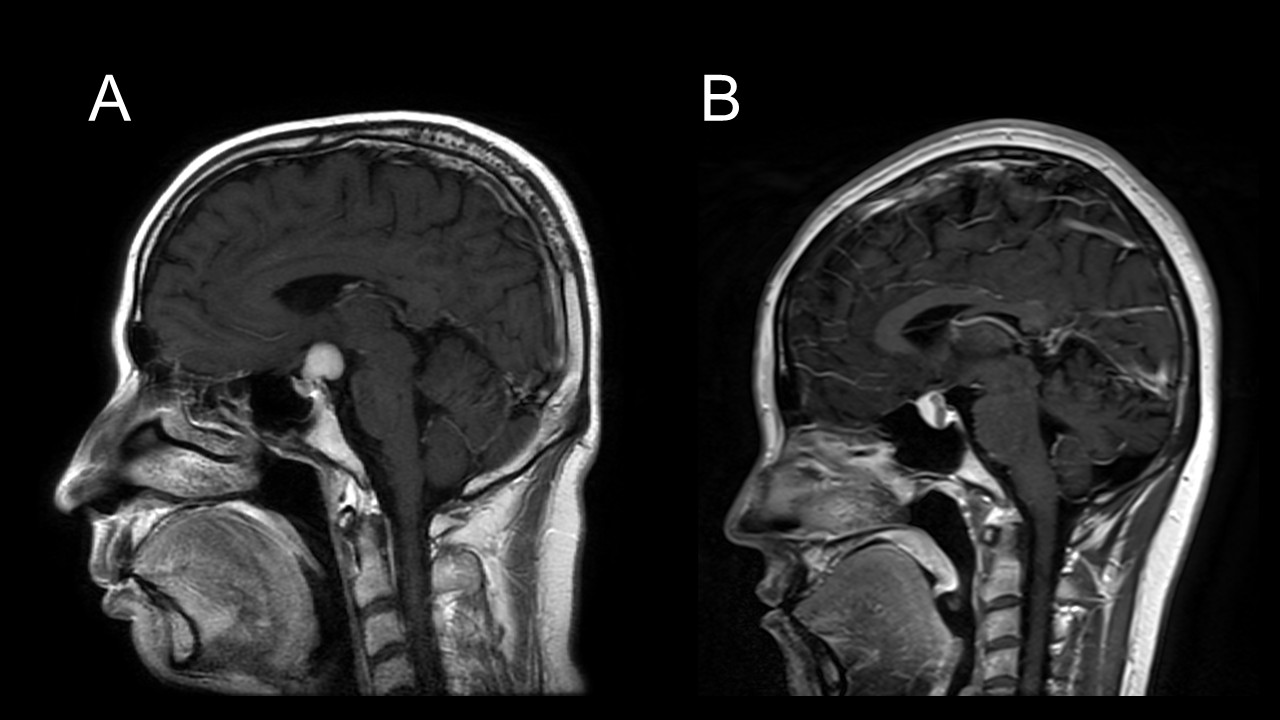


Supplementary Figure 1. (A) Correctly classified PC with typical imaging features. (B) Misclassified PC with atypical imaging features. This case was also radiologically misinterpreted as “PitNET with apoplexy”.
